# Significance of CD8^+^ T cell infiltration-related biomarkers and the corresponding prediction model for the prognosis of kidney renal clear cell carcinoma

**DOI:** 10.18632/aging.203584

**Published:** 2021-10-04

**Authors:** Yuan Tian, Yumei Wei, Hongmei Liu, Heli Shang, Yuedong Xu, Tong Wu, Wei Liu, Alan Huang, Qi Dang, Yuping Sun

**Affiliations:** 1 Somatic Radiotherapy Department, Shandong Second Provincial General Hospital, Shandong Provincial ENT Hospital, Jinan 250023, Shandong, P.R. China; 2 Head and Neck Radiotherapy Department, Shandong Provincial ENT Hospital, Cheeloo College of Medicine, Shandong University, Jinan 250023, Shandong, P.R. China; 3 Radiotherapy Oncology Department, The First Affiliated Hospital of Shandong First Medical University and Shandong Provincial Qianfoshan Hospital, Shandong Key Laboratory of Rheumatic Disease and Translational Medicine, Shandong Lung Cancer Institute, Jinan 250014, Shandong, P.R. China; 4 Endocrinology Department, Shandong Provincial Qianfoshan Hospital, The First Hospital Affiliated with Shandong First Medical University, Jinan 250014, Shandong, P.R. China; 5 Department of Oncology, Jinan Central Hospital, The First Hospital Affiliated with Shandong First Medical University, Jinan 250013, Shandong, P.R. China; 6 Department of Radiotherapy Oncology, Shandong Cancer Hospital and Institute, Shandong First Medical University and Shandong Academy of Medical Sciences, Jinan 250012, Shandong, P.R. China; 7 Phase I Clinical Trial Center, Shandong Cancer Hospital and Institute, Shandong First Medical University and Shandong Academy of Medical Sciences, Jinan 250012, Shandong, P.R. China

**Keywords:** CD8+T, cell infiltration, biomarkers, prognosis, KIRC

## Abstract

Cytotoxic T cells expressing cell surface CD8 played a key role in anti-cancer immunotherapy, including kidney renal clear cell carcinoma (KIRC). Here we set out to comprehensively analyze and evaluate the significance of CD8^+^ T cell-related markers for patients with KIRC. We checked immune cell response in KIRC and identified cell type-specific markers and related pathways in the tumor-infiltrating CD8^+^ T (TIL-CD8T) cells. We used these markers to explore their prognostic signatures in TIL-CD8^+^ T by evaluating their prognostic efficacy and group differences at various levels. Through pan-cancer analysis, 12 of 63 up-regulated and 162 of 396 down-regulated genes in CD8+ T cells were found to be significantly correlated with the survival prognosis. Based on our highly integrated multi-platform analyses across multiple datasets, we constructed a 6-gene risk scoring model specific to TIL-CD8T. In this model, high TIL-CD8 sig score was corresponding to a higher incidence frequency of copy number variation and drug sensitivity to sorafenib. Moreover, the prognosis of patients with the same or similar immune checkpoint gene levels could be distinguished from each other by TIL-CD8 sig score.

## INTRODUCTION

Kidney cancer is among the ten most commonly occurring malignant tumors in both men and women and is well known as the most deadly urinary tract cancer. Even with active surgical treatment, most patients will inevitably die from tumor recurrence and metastasis [1–4]. According to the latest statistics from the United States, the incidence of kidney cancer ranks 6th among male and 9th among female cancer patients [1, 2]. The total number of new kidney cancer patients is expected to reach 76,000 in 2021 [2]. Kidney renal clear cell carcinoma (KIRC) is the most common pathological type, accounting for about 70% of all [5, 6]. 80% of patients with advanced renal cancer who had received active surgical resection treatment, survived less than 2 years [5, 6]. Recent studies have reported multiple genes that are possibly involved in the occurrence and development of kidney cancer, such as VHL, MET and FLCN. U.S. Food and Drug Administration (FDA) has approved 7 drugs targeting the VHL signaling pathway, but the guiding significance for overall clinical outcome and the survival of kidney cancer patients were very limited. The lack of efficient treatment options is urging for more basic research on the development and prognosis prediction of KIRC in order to have a more thorough understanding of the pathogenesis at a molecular level, so as to provide some novel targets for therapeutic interventions [7, 8].

In recent years, large comprehensive patient data cohorts have become available leading to plenty of important clinical targets identified in various cancer types [9–12]. New tumor-related predictive indicators have also been discovered through bioinformatics analysis techniques [13–15]. Here we set out to thoroughly analyze datasets related to KIRC to explore and dissect the important role of CD8 (+) T cells in tumor immune infiltration and immunotherapy [16–20]. Our findings would be helpful for clarifying the significance of CD8 (+) T cell related markers in KIRC.

## RESULTS

### De-batch effect of immune cells in GEO chips

**Figure 1:**
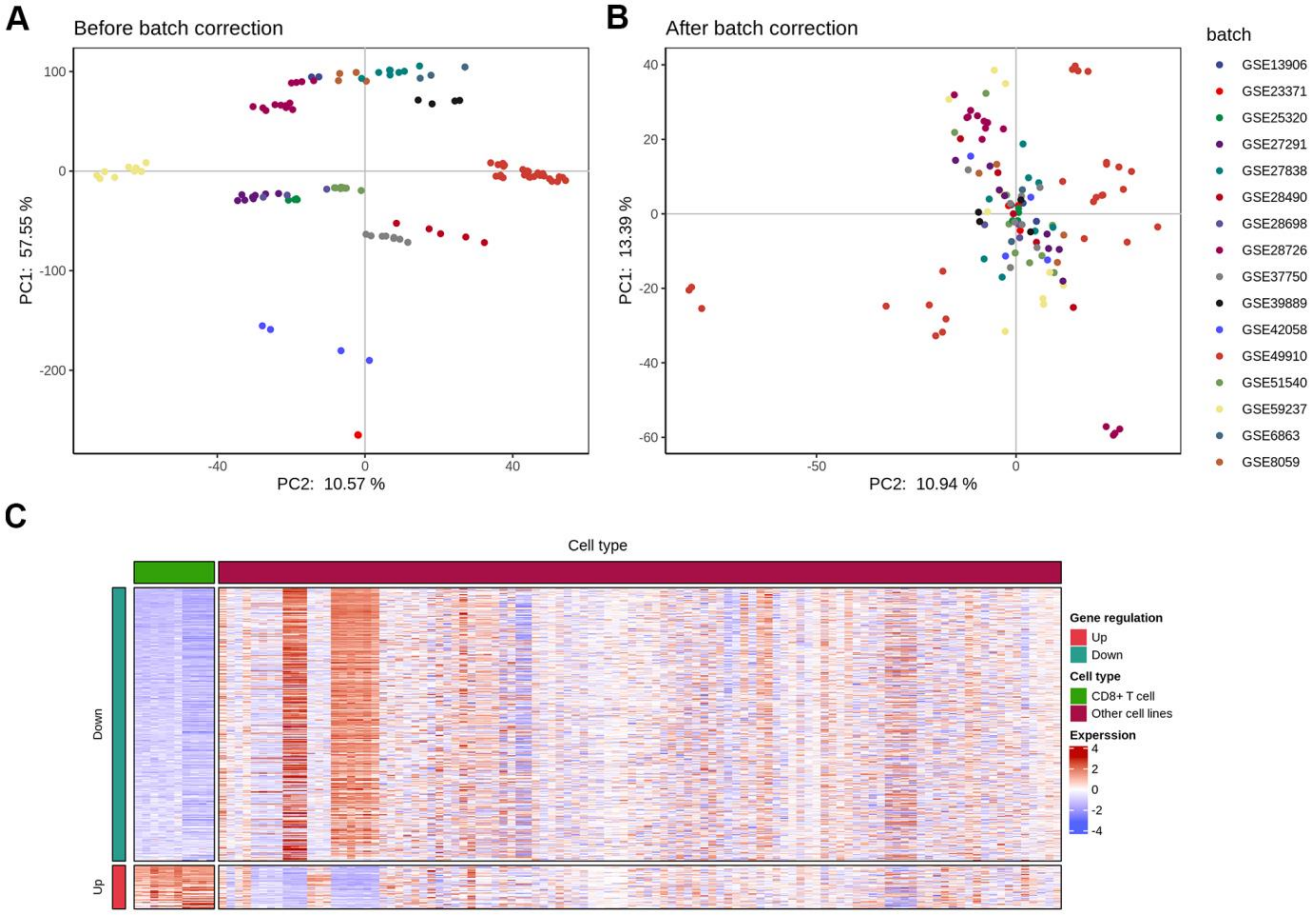
**Elimination of inter-study bias in immune cell chip data.** The batch effect correction of each chip was completed by the inSilicoMerging software package of Bioconductor. (**A**) Principal component analysis (PCA) analysis of each chip data before batch effect was removed; (**B**) PCA analysis of each chip data after the batch effect was removed; (**C**) The differential expression (|logFC|>1 and p<0.05) gene heat map of CD8 T cells and other immune cells.

To compare immune cell response in KIRC and identify cell type-specific markers and related pathways in the tumor-infiltrating CD8^+^T (TIL-CD8T) cells, transcriptome data was collected from immune cells across multiple studies. The expression results of the immune cell chip data was downloaded from the GEO database (Table 1), and the data underwent an inter-study bias correction by the inSilicoMerging software package. The chip data distribution before and after batch effect calibration, were displayed in (Figure 1A, 1B), respectively.

**Table 1:** Summary of immune cell types in the chip.

**Population**	**Data set**	**Cell numbers**
B cell activated	GSE28490, GSE49910	9
CD4 T cell activated	GSE28726, GSE49910	7
CD4 T cell resting	GSE28726	4
CD8 T cell activated	GSE49910	6
CD8 T cell resting	GSE49910	4
Dendritic cells activated	GSE59237	4
Dendritic cells resting	GSE59237	6
Eosinophils	GSE28698	3
Immature dendritic cells	GSE23371, GSE6863	6
Mast cells activated	GSE25320	4
Monocytes	GSE49910	6
Myeloid dendritic cells	GSE42058	4
Neutrophils	GSE39889, GSE49910	7
NK activated	GSE27838, GSE8059	11
NK resting	GSE8059	1
NKT activated	GSE28726	6
Plasmacytoid dendritic cells	GSE37750	8
T gamma delta	GSE13906, GSE27291	10
T helper 17	GSE51540	9

### Screening for specific marker genes of TIL-CD8 T cells

To explore CD8 T cell response upon tumor infiltration and identify a set of genes that were unique markers for TIL-CD8T cells, a systematic transcriptome analysis across multiple immune cells was performed by the Seurat's Findmarkers function. Genes with significant differential expressions (|logFC|>1 and p<0.05) were considered to be specifically expressed by TIL-CD8 T cells. In total of 63 genes were found to be up-regulated in CD8 T cells, while 396 genes were down-regulated (Figure 1C). As the next step, an analysis was put into practice to clarify whether these genes were correlated with patient survival data. Through the pan-cancer analysis, 12 of 63 up-regulated and 162 of 396 down-regulated genes were found to be significantly correlated with the survival prognosis (Supplementary Table 1). Annotations for all cell lines were provided in (Supplementary Material 1), while the details of all cell lines were summarized in (Supplementary Material 2).

### GO and KEGG enrichment analyses of specific marker genes of TIL-CD8 T cells

In order to further investigate the biological function of TIL-CD8 T cell-specific marker genes, GO enrichment analysis and KEGG pathway enrichment analysis on the differentially expressed genes were performed. Specifically, the results of the top significant enrichment analyses for molecular function (MF), biological process (BP) and cellular component (CC) (Supplementary Figure 1A–1C) were evaluated, respectively. Neutrophil activationa and degranulation as well as T cell activation were included in the top biological GO categories. The enrichment analyses results of KEGG pathway were shown in (Supplementary Figure 1D), which chemokine signaling pathway was listed as the top hit.

### TIL-CD8T-related prognostic factors in KIRC screened by univariate cox regression analysis

Then, the prognostic value of the identified marker genes was assessed. After filtering out patients with no survival information in the TCGA-KIRC cohort, a total of 307 samples with clinical survival data were further analyzed. Through univariate Cox regression analysis, 38 prognostic factors related to KIRC TIL-CD8T were obtained. The maximum selection rank sum statistics of the R software package "maxsat" was used to determine the cutoff value of the prognostic factors.

**Figure 2:**
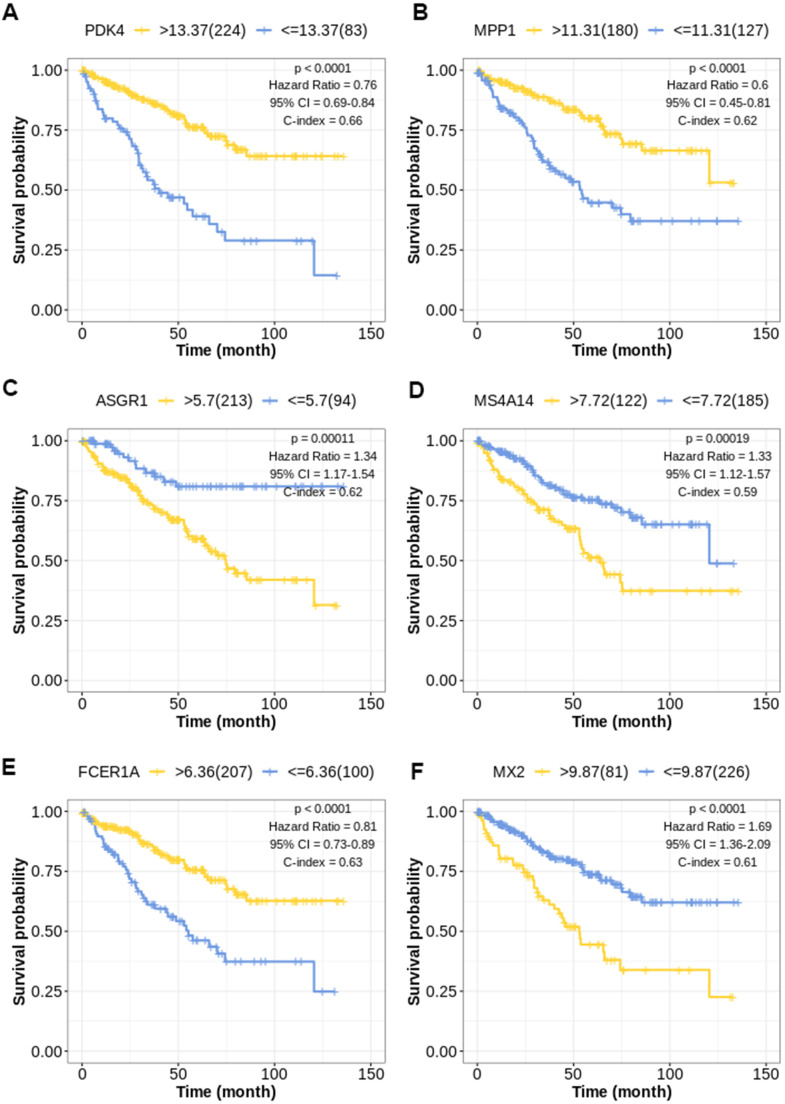
**Kaplan-Meier survival curve for TIL-CD8T-related prognostic factors.** The abscissa axis represents survival time; the ordinate axis represents survival probability. The survival curves of different colors represent different expression status of related genes. (**A**) Kaplan-Meier survival curve of PDK4 (P < 0.0001): Hazard Ratio=0.76, 95%CI [0.69, 0.84]; C-index= 0.66. (**B**) Kaplan-Meier survival curve of MPP1 (P < 0.0001): Hazard Ratio=0.60, 95%CI [0.45, 0.81]; C-index= 0.62. (**C**) Kaplan-Meier survival curve of ASGR1 (P = 0.00011): Hazard Ratio=1.34, 95%CI [1.17, 1.54]; C-index= 0.62. (**D**) Kaplan-Meier survival curve of MS4A14 (P = 0.00019): Hazard Ratio=1.33, 95%CI [1.12, 1.57]; C-index= 0.59. (**E**) Kaplan-Meier survival curve of FCER1A (P < 0.0001): Hazard Ratio=0.81, 95%CI [0.73, 0.89]; C-index= 0.63. (**F**) Kaplan-Meier survival curve of MX2 (P < 0.0001): Hazard Ratio=1.69, 95%CI [1.36, 2.09]; C-index= 0.61.

The Kaplan-Meier curve of top 6 factors (PDK4, MPP1, ASGR1, MS4A14, FCER1A, MX2) was shown in (Figure 2A–2F), and the cutoff values of the prognostic factors were shown in (Supplementary Table 2).

### Construction of a TIL-CD8T-related tumor gene risk scoring model for KIRC (6 genes)

Through univariate Cox regression analysis, 38 TIL-CD8T-related genes were found to be significantly related to the prognosis of patients with KIRC (Supplementary Figure 2A). Then, the "cv.glmne" function of the R "glmnet" package was used for LASSO regression implement to further screen prognostic-related genes. The family parameter was set as "cox" and nfold=10. According to the lambda, the complexity of the model was optimized by LASSO regression. The "cv.glmne" analysis result contained two optimization models, one was "lambda.min" and the other was “lambda.1se”. The lambda between these two values was considered appropriate. The model constructed by "lambda.1se" was the simplest, which the number of involved genes was few, while the accuracy of "lambda.min" was higher. Here, 13 prognostic-related genes in the LASSO model corresponding to "lambda.min" was collected for follow-up analyses.

After the above analysis, 13 genes related to the prognosis of KIRC patients were identified (Supplementary Figure 2B, 2C). Then, based on these 13 genes, a multivariate Cox regression analysis was used for the TIL-CD8T risk scoring model construction. During the construction process, both the prediction accuracy and the simplicity of the model was taken into account. The "stepAIC" function in the "MASS" software package was used to screen the 13-variable factors in the multivariate Cox regression model. The parameter was set as "direction=both", "both" corresponded to the stepwise regression analysis method, starting from no predictor variables, and then adding the most contributory (calculate the "AIC" value according to the model after adding the variables, and select the smaller "AIC" value Variables) predictor variables in turn (such as forward selection). After adding each new variable, any variables that no longer provided improved model fit (the AIC value of the model could not be reduced after the variable was enrolled) would be deleted. Based on our multi-step iterative analyses, the following risk scoring model was constructed, which involved 6 genes:

TILCD8Sigscore = (-0.121 * PDK4) + (-0.492 * MPP1) + (0.22 * ASGR1) + (0.176 * MS4A14) + (-0.184 * FCER1A) + (0.5 * MX2)

### Verification of the TIL-CD8T-related tumor gene risk scoring model by external data

**Figure 3:**
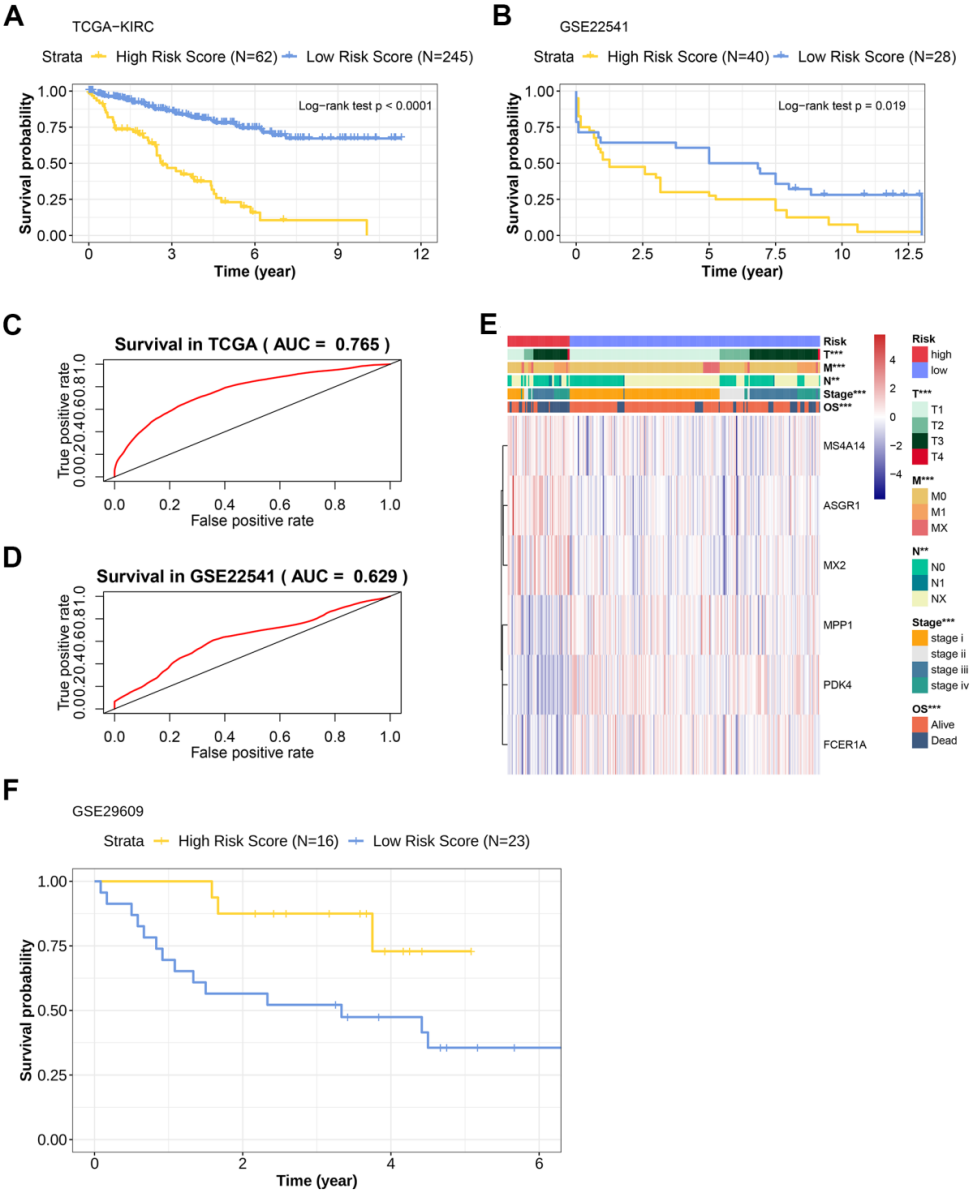
**Prognostic efficacy evaluation of risk scoring model and external data verification.** (**A**) Kaplan-Meier overall survival curve of patients in the TCGA-KIRC cohort. The abscissa axis represents survival time; the ordinate axis represents survival probability. The survival curves of different colors represent different risk score subgroups. (**B**) Kaplan-Meier overall survival curve of patients in the GSE22541 cohort. The abscissa axis represents survival time; the ordinate axis represents survival probability. The survival curves of different colors represent different risk score subgroups. (**C**) The predictive efficiency of the risk scoring model in the TCGA-KIRC cohort (AUC= 0.765). The abscissa axis represents false positive rate; the ordinate axis represents true positive rate. (**D**) The predictive efficiency of the risk scoring model in the GSE22541 cohort (AUC= 0.629). The abscissa axis represents false positive rate; the ordinate axis represents true positive rate. (**E**) Heatmap representation of the expression levels of genes included in the KIRC scoring model of the low and the high-risk groups, and the distribution of clinicopathological characteristics in the low and high-risk groups. (**F**) Kaplan-Meier overall survival curve of patients with KIRC in the GSE29609 cohort. The abscissa axis represents survival time; the ordinate axis represents survival probability. The survival curves of different colors represent different risk score subgroups.

In order to verify the prediction efficiency of the above model in an independent data set, the model was used to calculate the risk scores (TIL-CD8T-Sigscore) in KIRC sample chip data (GSE22541, GSE29609 and TCGA-KIRC). The disease-free survival (DFS) in GSE22541 was used as an index for clinical benefit evaluation. It was found that the survival prognosis of patients with low TIL-CD8T-Sigscore in these data sets was significantly better than those with high risk scores (Figure 3A–3B). The AUC of the 5-year OS, predicted by the TCGA data, was 0.765 (Figure 3C), while the AUC of the 5-year survival time predicted by the GSE22541 data was 0.629 (Figure 3D). In contrast, the survival prognosis of patients with low risk score of GSE29609 was worse than those of the high risk score group (Figure 3F). In order to further assess the correlation between clinical information and the individual expression of prognostic factors, this information was visualized in a heatmap. As shown in (Figure 3E), high risk group corresponded to the high mortality. This trend could also be found in TNM staging (Figure 3E).

### Genes expression profile included in the scoring model across KIRC cell lines

The expression profile of the 6 genes risk scoring model was compared between KIRC cell line and CD8 T cell data set, and then the Wilcoxon rank sum test was used to evaluate the differential expression significance of these genes in the CD8 T and KIRC cell lines. However, no significant expression difference or specificity corresponding to certain KIRC cell lines was found (Supplementary Figure 3A–3F).

### Single-sample gene set enrichment analysis (ssGSEA) of immune cell infiltration level in TIL-CD8T Sig score group

Next, the immune infiltration levels in the high and low TIL-CD8T Sig score groups were further evaluated. ssGSEA was used in 19 immune cell subgroups. Dendritic and mast cells were found to be enriched in the low score group, while Act.B, Act.dendritic, Act.CD4 T, Act.CD8 T, Monocyte, Th17, NK.T, NK, Eosinophil, Plas.dendritic and Neutrophil cells were hyper-infiltrated in the high score group (Supplementary Figure 4A).

### The landscape analysis of mutation and copy number variation (CNV) in high and low TIL-CD8Sig score groups

**Figure 4:**
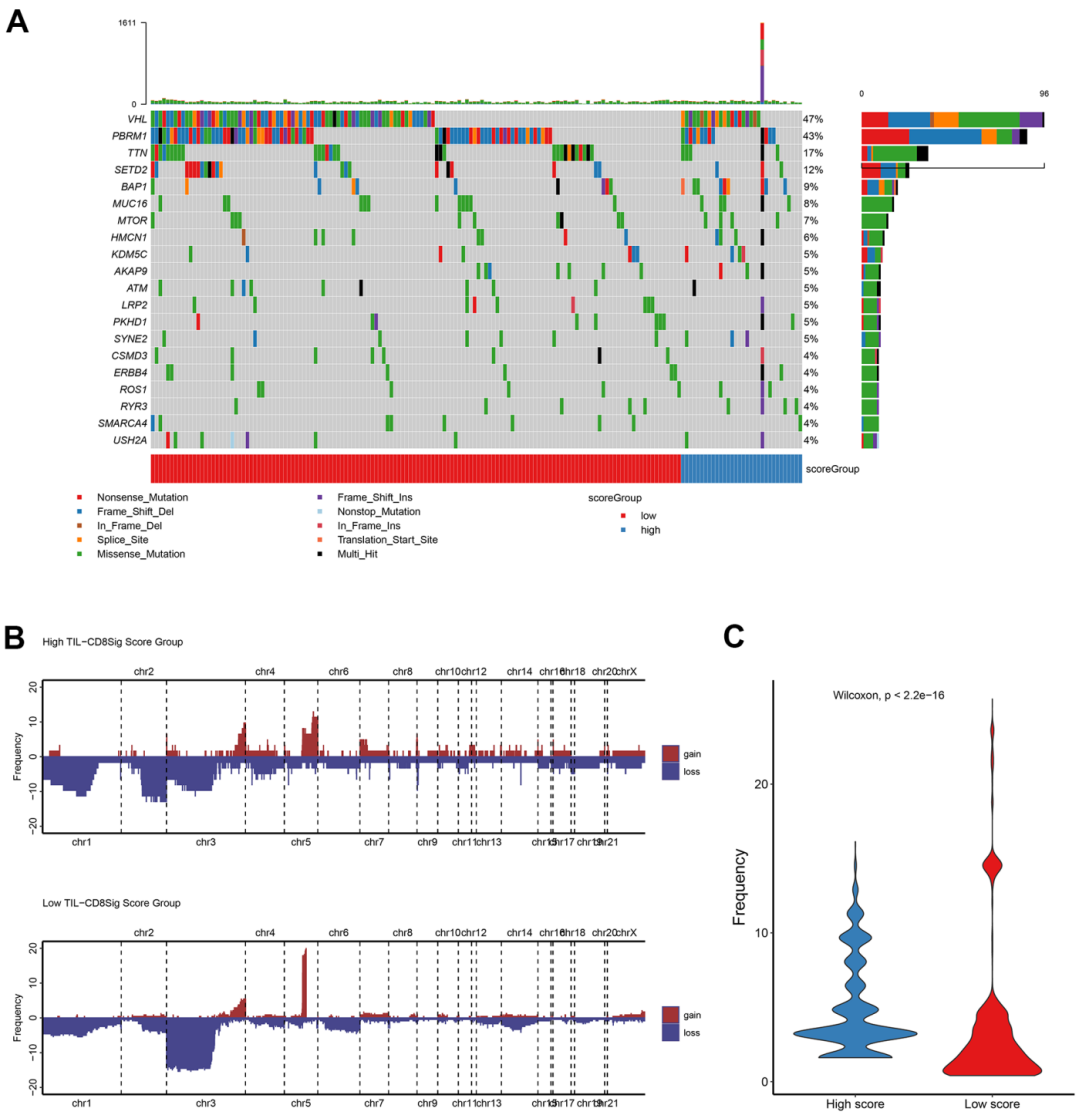
**Genetic landscape analysis of mutation and copy number variation (CNV) in high and low TIL-CD8Sig score groups.** (**A**) Mutation waterfall graph of different TIL-CD8Sig score subgroups in the TCGA-KIRC cohort. (**B**) CNV spectrum of different TIL-CD8Sig score subgroups in the TCGA-KIRC cohort. Different colors represent different CNV types (gain or loss); the abscissa axis represents chromosome locus; the ordinate axis represents CNV frequency. (**C**) The difference of mutation frequency in different TIL-CD8 Sig score subgroups. The abscissa axis represents different scoring groups; the ordinate axis represents mutation frequency.

In order to clarify the correlation between TIL-CD8 Sig score and gene mutations, the R software package maftools was used to deal with the publicly available mutation annotation format (maf) files (Figure 4A), and evaluate the relationship between different TIL-CD8Sig scores and CNV (Figure 4B). It was found that the gene mutations and CNV status of patients in different TIL-CD8Sig score groups were different, and the CNV frequency was significantly different from each other (Figure 4C).

### Differences of the immunotherapy efficacy in patients with different TIL-CD8 Sig scores predicted by tumour immune dysfunction and exclusion (TIDE) score

In order to investigate the differences of the immunotherapy efficacy in patients with different TIL-CD8 Sig scores, the TIDE score was used for prediction practice. The TIDE method could integrate the expression characteristics of T cell dysfunction and rejection to simulate tumor immune escape, and then adopt untreated tumor data to predict the clinical response of immune checkpoint blockade (ICB). The TIDE online analysis website (http://tide.dfci.harvard.edu/) was used to calculate the TIDE score, and evaluate the difference among different TIL-CD8Sig score groups. However, no significant difference was found in the two groups (Supplementary Figure 4B).

### The drug resistance (Sorafenib) prediction in TIL-CD8Sig score group

Sorafenib represents one of the standard treatment options for patients with metastatic renal cell carcinoma. However, highly treatment resistant is common in KIRC. The mechanism of drug resistance was still not well understood. To reveal the relationship between TIL-CD8 Sig score and drug resistance in patients with KIRC, the R software package pRRophetic was adopted for predicting the sensitivity of Sorafenib. Through analysis, it was found that the sensitivity of Sorafenib (Supplementary Figure 4C) in the low-scoring group was significantly different from that of the high-scoring group.

### Independence verification of prognostic factors

To further verify the predictive value of TIL-CD8Sig score as a prognostic factor, Univariate and Multivariate Cox regression analyses were used to deal with the data of TCGA-KIRC and GSE22541. Through these analyses, it was found that the 6-gene TIL-CD8 Sig score model could be used as an independent prognostic factor in both TCGA and GSE22541 (Supplementary Figure 5A–5D).

### Construction of nomogram for the prediction of overall survival rate

**Figure 5:**
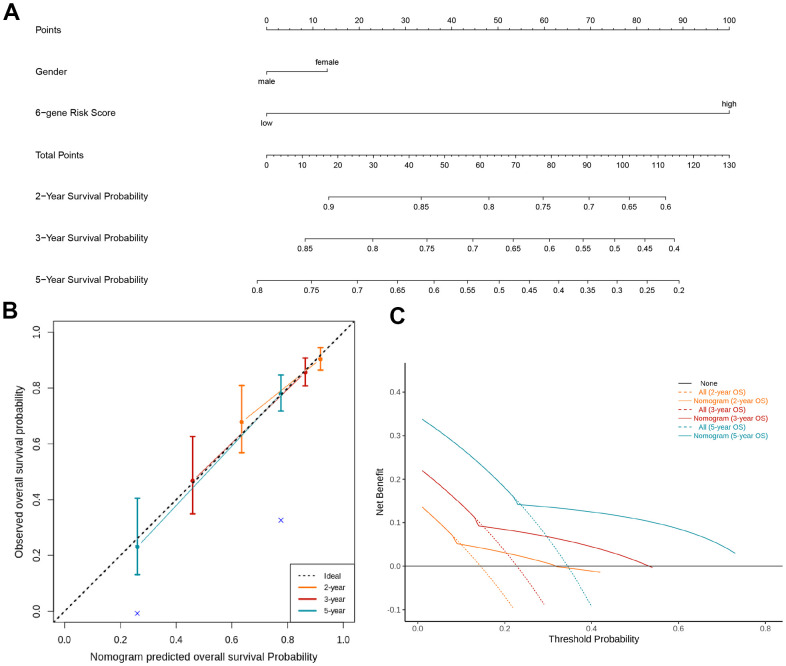
**Nomogram and decision analysis curve for predicting the overall survival of KIRC patients.** (**A**) Combining the TIL-CD8Sig score of the TCGA-KIRC cohort data and clinical pathological risk factors to predict the 2-year, 3-year and 5-year overall survival probability. (**B**) According to the consistency of the prediction and observation results, the correction map of the nomogram was drawn. The performance of the nomogram was shown by the chart relative to the dotted line, which the dotted line indicated a perfect forecast. (**C**) Nomograph's decision analysis curve. None: Hypothetical events will not occur in any patients (horizontal solid line); All: Hypothetical events will occur in all patients (dotted line), the expected net income based on the nomogram prediction under different threshold probabilities was displayed by it.

Nomograms had been previously shown as a reliable alternative tool that could help clinicians make individual predictions for survival. By integrating the TIL-CD8Sig score groups and clinicopathological risk factors, a nomogram for predicting the overall survival rate of TCGA-KIRC was constructed (Figure 5A). Through the standard curve diagram, it was found that the performance of the nomogram was equivalent to that of the ideal model (Figure 5B). Decision curve analysis (DCA) was used to quantify the clinical utility of it. For the overall survival probabilities of 2, 3, and 5 years, the decision curve showed that the nomogram could provide a better net benefit than the alternatives (Figure 5C).

### Analyses of the combination of TIL-CD8 Sig score and immune check sites expression as the prognostic factors in KIRC

**Figure 6:**
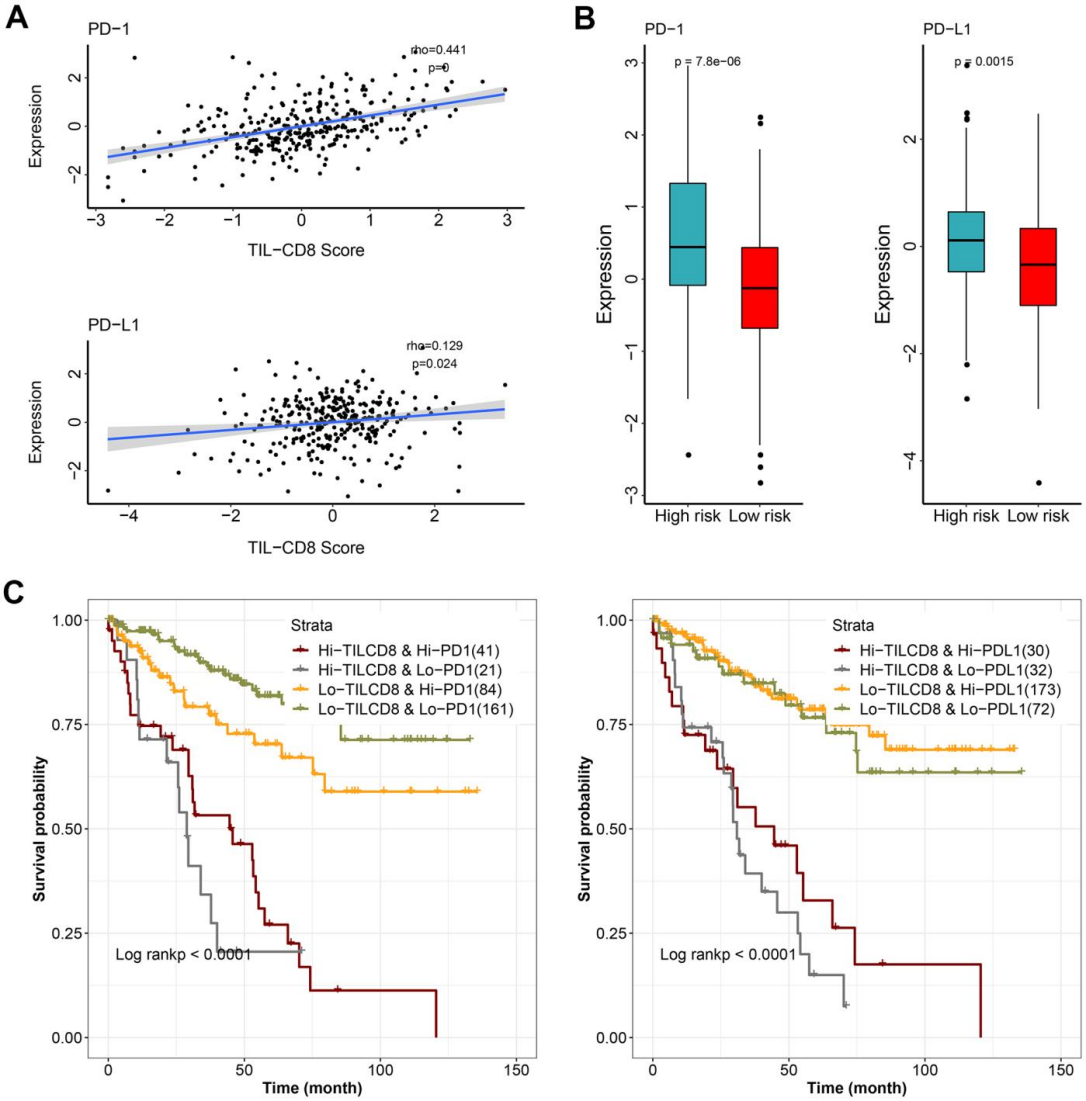
**The effect of TIL-CD8Sig and immune checkpoint gene expression on patient survival.** (**A**) The relationship between TIL-CD8Sig and the expression of immune checkpoint genes (PD-1 and PD-L1). The abscissa axis represents the TIL-CD8Sig Score, and the ordinate axis represents the expression level of PD-1/PD-L1. (**B**) Box plot of the expression distribution of immune checkpoint genes (PD-1 and PD-L1) in the high and low risk groups of TIL-CD8Sig. The abscissa axis represents different risk groups; the ordinate axis represents the expression level of PD-1/PD-L1. (**C**) Kaplan-Meier survival curve of OS in four groups of patients stratified by TIL-CD8Sig and immune checkpoint gene expression. The abscissa axis represents survival time; the ordinate axis represents survival probability.

Immune checkpoint molecules, such as PD-1 and PD-L1, had been identified as crucial regulators of the immune response. However, the prognostic significance of these immune checkpoint molecules was still controversial. We wanted to further investigate the relationship between TIL-CD8 Sig score and the PD-1/PD-L1 expression levels. As shown in (Figure 6A), the TIL-CD8 Sig score was significantly correlated with both PD-1 and PD-L1 (PD-1: spearman correlation coefficient rho= 0.441, P <0.001; PD-L1: spearman correlation coefficient rho=0.129, P=0.024). To further evaluate the expression patterns of immune checkpoint genes in different TIL-CD8Sig score groups, it was found that the PD-1 (Wilcoxon rank sum test P<0.001) and PD-L1 (Wilcoxon rank sum test P<0.01) expression levels in the high-risk group were significantly higher than those of the low-risk group (Figure 6B).

Finally, to further clarify the impact of the interaction between TIL-CD8Sig score and immune checkpoint genes on survival, the patients were divided into 4 groups based on the combination of TIL-CD8Sig score (high/low) and immune checkpoint genes (high/low). Survival comparisons were put into practice in the 4 groups, and the results were displayed in (Figure 6C). The comparison results showed that the prognosis of patients with the same or similar immune checkpoint gene levels could be distinguished from each other by TIL-CD8Sig score (PD-1: log rank *P* <0.0001; PD-L1: log rank *P* = <0.0001) (Figure 6C).

## DISCUSSION

Previous studies have raised that CD8+ T cell infiltration can play an important role in anti-tumor immunotherapy [18–22]. Indeed, several immunotherapy targets had been identified in CD8+ T cells [23], suggesting that CD8T should be considered for other anti-cancer therapies. Here we aimed to clarify the significance of CD8+ T cell infiltration related markers for KIRC patients.

Our work relied on processing data from various database. In order to reduce the bias and heterogeneity of the data source, the most commonly used TCGA database for downstream bioinformatics analysis was given priority [24, 25]. After ensuring that de-batch effect and inter-study bias had been efficiently removed by calibration, and the distribution of GEO chip data had became uniform, a large sets of data from GEO were collected for further verification (Table 1). 63 genes were found to be up-regulated in CD8 T cells, while 396 genes were found to be down-regulated (Figure 1C). Through pan-cancer analyses, 12 of 63 up-regulated and 162 of 396 down-regulated genes were found to be significantly correlated with the survival prognosis (Supplementary Table 1), which were rarely reported in previous studies [18, 26]. These dysregulated genes, identified in our analyses, might be potential biomarkers for the prognosis of KIRC [26, 27].

Neutrophils played an important role in cancer [28], and were reported to be related to tumor progression and immunity [29]. After biological process (BP) enrichment analyses of specific marker genes in TIL-CD8 T cells, it was found that neutrophil-associated terms, such as neutrophil activation and degranulation, were enriched (Supplementary Figure 1A). This suggested that TIL-CD8TSig might participant in cancer development by regulating the activity of neutrophils [28, 29]. This correlation further reinforced the possibility that TIL-CD8TSig could be used as a prognostic indicator. For molecular function (MF) and cellular component (CC), no enrichment analyses results with such obvious commonality were found (Supplementary Figure 1B, 1C). KEGG pathway enrichment analyses were found to be mainly enriched in tumor-related signal pathways (Supplementary Figure 1D) [30–36]. Chemokine signaling pathway, ranked as the first one, had been frequently reported in cancers and immunotherapy [31, 37], but it was rarely reported in KIRC. Similar status could also be seen in other pathways (Supplementary Figure 1D) [31, 36]. While our analyses represented a significant conclusion from our current knowledge, the mechanism of TIL-CD8TSig involved signal pathways in KIRC were needed to be further investigated [30–36].

Based on our highly integrated multi-platform analyses across multiple datasets, the risk scoring model specific to TIL-CD8T was constructed. LASSO Cox regression, widely used in cancer research for predicting model construction, was adopted in our study [38–40]. Based on LASSO and TCGA-KIRC, the 6-gene risk scoring model corresponded to TIL-CD8T was constructed (Figure 2 and Supplementary Figure 2), which was further verified by GEO data cohorts (Figure 3). Those 6 genes in the model (PDK4, MPP1, ASGR1, MS4A14, FCER1A and MX2), rarely reported in cancers [41–44], were found to be significantly related to the prognostic survival of KIRC patients (Figure 2). In the model effectiveness evaluation, similar survival-related trend could also be found (Figure 3A, 3B, 3F). However, the survival trend in GSE29609 was contrary to those of TCGA-KIRC and GSE22541 (Figure 3A, 3B, 3F). In order to further explore the reasons for the failure of the model prediction in GSE29609, the primary data set was carefully checked. It was found that the limited number of patients and the high degree of heterogeneity among individuals might be the main disadvantages that led to the failure of the model prediction (Figure 3F). The ROC curve showed that the risk scoring model had a good predictive effect in TCGA-KIRC and GSE22541 (Figure 3C, 3D). Overall, it could be concluded that a higher risk score in our model corresponded to a higher mortality rate, which also indicated that our risk scoring model had a better predictive efficiency for the survival prognosis of KIRC patients (Figure 3E).

Further analyses showed that there was no significant difference in the expression of genes contained in the risk scoring model between the KIRC cell lines and CD8 T cell data sets (Supplementary Figure 3A–3F), which further confirmed the universality of the model applicability. ssGSEA analyses showed that much more immune cells were enriched and activated in the high TIL-CD8T Sig score group (Supplementary Figure 4A). Moreover, different TIL-CD8Sig scores corresponded to different gene mutations and CNV status (Figure 4A, 4B), and there was a significant difference in CNV frequency between the two groups (Figure 4C). Regardless of the group, the VHL gene was still the most frequently altered gene (Supplementary Figure 4B), which was consistent with previous studies in KIRC [5, 45]. This also indirectly verified the reliability of our analysis results.

In our analyses, we also wanted to explore the underlying causes of drug resistant in KIRC, which was frequently reported in previous reports [46, 47]. Sorafenib is one of the most often used drug for metastatic KIRC [46, 47]. Strikingly, it was found that IC50 of sorafenib was significantly different between high and low TIL-CD8T Sig score groups (Supplementary Figure 4C). These findings suggested that the model could be used for evaluating the sensitivity of sorafenib based on the TIL-CD8T Sig score in future clinical work. This further expanded the scope of potential application of our research results.

Moreover, our univariate and multivariate Cox regression analyses confirmed that the 6-gene TIL-CD8 Sig scoring prediction model could be used as an independent prognostic factor in both the TCGA-KIRC and GSE22541 data cohorts (Supplementary Figure 5). The results of the nomogram and decision curve analysis also suggested that the prediction model had a good predictive efficiency for the prognostic survival of patients (Figure 5). Furthermore, TIL-CD8T Sig score could be used for distinguishing the survival prognosis of patients with the same or similar immune checkpoint gene levels (Figure 6), which would be helpful for us to choose the use of PD-1/PD-L1 drugs [47, 48].

Unlike previous reports [49, 50], more indicators were analyzed and evaluated in this study. In the process of analyses, based on the data of TCGA, as many data from external databases as possible were used for further verification. Different from the previous co-expression network and protein-protein interactions network analysis [26], the 6 prognostic-related genes predicting model was constructed and further well verified, which was also the main innovation and attempt of this research. These advantages strengthened the significance of our work.

Although different data sets were used to mutually verify the analyses conclusions, the limitations of the study should be acknowledged. The obvious limitation of this hypothetical study was the fact that there was no laboratory based real experiments or any clinical study were conducted. However, due to the rigorous design and analyses process, it was believed that our analyses results still had some important guiding significance for clinical applications.

## CONCLUSIONS

Low levels of TIL-CD8T Sig score was associated with a better prognosis in KIRC patients. The 6-gene TIL-CD8 Sig scoring prediction model might be a good choice for the prediction of the prognostic survival, immunotherapy options and sorafenib drug sensitivity in KIRC patients.

## MATERIALS AND METHODS

### Published data sets

The transcriptome data and related clinical information of patients with KIRC were obtained through Xena (http://xena.ucsc.edu/). 307 KIRC patient data sets containing transcriptome and clinical information were collected from the TCGA-KIRC sample cohort. All clinical data information of TCGA were provided in Supplementary Material 3. 68 KIRC patient datasets with transcriptome and clinical information were obtained through the GEO database (https://www.ncbi.nlm.nih.gov/geo/, GSE22541). Data including a total of 65 KIRC cell lines were extracted through Genomics of Drug Sensitivity in Cancer (GDSC) (https://www.cancerrxgene.org/) and Cancer Cell Line Encyclopedia (CCLE) (https://portals.broadinstitute.org/ccle/). Among them, 32 KIRC cell lines were picked up through the GDSC database, while 33 KIRC cell lines were collected from the CCLE database.

Immune cells related transcriptome data were downloaded from the GEO database, including GSE42058, GSE49910, GSE51540, GSE59237, GSE6863, GSE8059, GSE13906, GSE23371, GSE25320, GSE27291, GSE27838, GSE28490, GSE28698, GSE28726, GSE37750, and GSE39889. The cell types corresponding to every chips were summarized in (Table 1).

### Data preprocessing

For the data of immune cells from multiple chips, the Bioconductor package inSilicoMerging was used for inter-study bias correction. The data of the TCGA-KIRC cohort was standardized by using log2 (FPKM+1).

### Screening for specific marker genes in tumor infiltrating CD8+ T lymphocytes

Tumor infiltrating CD8 T cell gene signature (TIL-CD8TSig) was established as follows: 1) The differential expression analyses of CD8 T cells and other immune cells were performed by the Seurat's Findmarkers function of R package. Genes with significant differential expressions (|logFC|>1 and p<0.05) were considered to be specifically expressed by TIL-CD8 T cells; 2) TIL-CD8T genes related to the prognosis of KIRC patients were screened by the univariate Cox regression analysis. Then, the optimal combination of forward and backward variables of the multivariate Cox regression model was used for the screening; 3) Finally, the expression value of the prognostic factor weighted by the multivariate Cox regression coefficient was converted into a risk score (TIL-CD8TSig score) for clinical application.

### Enrichment analysis of specific marker genes of TIL-CD8 T cells

In order to investigate the function of the specifically expressed genes of TIL-CD8 T cells, Gene Ontology (GO) and Kyoto Encyclopedia of Genes and Genomes (KEGG) enrichment analysis for specific marker genes were performed by the R package clusterProfiler. p-adjusted < 0.05 was considered as the threshold for significant enrichment.

### Construction of TIL-CD8T-related risk scoring model

A large number of TIL-CD8T factors related to prognosis were screened by the Least Absolute Shrinkage and Selection Operator (LASSO) regression model. Then, multivariate Cox regression analysis was performed for the prognostic factors optimized by the LASSO regression model, and the stepAIC function in the MASS (Modern Applied Statistics with S) package of the R software was used for stepwise regression performance to screen the model factors.

### Identification of TILs immune cell subsets

Single-sample gene set enrichment analysis (ssGSEA) was used to identify immune cells that were hyper-infiltrating in the tumor microenvironment. The degree of correlation was expressed by the Normalized Enrichment Score (NES).

### Pan-cancer analysis of CD8T-related genes in KIRC

All CD8T-related genes were used for pan-cancer analysis of differential gene expression and survival analysis by an online available tool (http://starbase.sysu.edu.cn/panCancer.php). All the R scripts involved in the above analysis were provided in Supplementary Material 4.

### Statement of ethics

Since this research does not involve the interaction with human subjects, no ethical issues were encountered, and no ethical approval was needed.

## Supplementary Material

Supplementary Material 1

Supplementary Material 2

Supplementary Material 3

Supplementary Material 4

Supplementary Figures

Supplementary Table 1

Supplementary Table 2
