# Supplementary material for: Significance of CD8+ T cell infiltration-related biomarkers and the corresponding prediction model for the prognosis of kidney renal clear cell carcinoma
Source: Aging (Albany NY). 2021 Oct 4;13(19):22912–33. doi: 10.18632/aging.203584 (PMC8544304; doi:10.18632/aging.203584)
Supplement: Supplementary Material 4 [file aging-13-203584-s004.doc]

**Supplementary Material 4. R Script for all the raw data.**

#### --------------------- 01.原始数据 ---------------------

setwd("/mnt/d/mywork/03-GAP-202004-2020/06-GAP201008002-肾透明细胞癌CD8+T免疫浸润细胞特有marker肿瘤分析/")

source("v1/packages.R")

runtime.path = "v1/runtime/01.原始数据"

result.path = "v1/results/01.原始数据"

dir.create(result.path,recursive = T)

dir.create(runtime.path,recursive = T)

#1 CD11c+ Myeloid Dendritic Cells

if(!file.exists(file.path(runtime.path,"GSE42058.rds"))){

GSE42058 = getGEO("GSE42058", GSEMatrix =TRUE, AnnotGPL=TRUE,destdir = file.path(runtime.path))

if (length(GSE42058) > 1) idx <- grep("GPL570", attr(GSE42058, "names")) else idx <- 1

GSE42058<- GSE42058[[idx]]

GSE42058 = GSE42058[,1:4] # 过滤HIV感染的细胞5：8

pData(GSE42058) = pData(GSE42058)[,1:2]

pData(GSE42058)$title = "Myeloid dendritic cells"

pData(GSE42058)$batch = "GSE42058"

# pData(GSE42058)$title %<>% as.character() %>% gsub(pattern = "(HIV )|(_\\d{3,4})|(uninfected )|(infected )",replacement = "")

colnames(pData(GSE42058))[1] = "cell.subtype"

GSE42058 = anno.GEO(GEO = GSE42058)

exprs(GSE42058) = log2(exprs(GSE42058))

saveRDS(GSE42058,file = file.path(runtime.path,"GSE42058.rds"))

}else(

GSE42058 = readRDS(file.path(runtime.path,"GSE42058.rds"))

)

#2 "Bcell" "Neutrophil" "CD8_Tcell" "CD4_Tcell" "Monocyte" "Erythroblast" "Bonemarrow&progenitors"

if(!file.exists(file.path(runtime.path,"GSE49910.rds"))){

GSE49910 = getGEO("GSE49910", GSEMatrix =TRUE, AnnotGPL=TRUE,destdir = file.path(runtime.path))

if (length(GSE49910) > 1) idx <- grep("GPL570", attr(GSE49910, "names")) else idx <- 1

GSE49910<- GSE49910[[idx]]

pData(GSE49910) = pData(GSE49910)[,1:2]

pData(GSE49910)$title %<>% as.character() %>%

# gsub(pattern = "(_Central_memory_)|(_effector_memory(_RA)?)|",replacement = "") %>%

gsub(pattern = "T_cell:CD4\\+:r\\d{1,2}",replacement = "CD4 T cell activated") %>%

gsub(pattern = "(T_cell:CD8\\+:r\\d{1,2})",replacement = "CD8 T cell activated") %>%

gsub(pattern = "T_cell:CD8\\+_naive:r\\d{1,2}",replacement = "CD8 T cell resting") %>%

gsub(pattern = "(.*) \\d{1,2}",replacement = "\\1") %>%

gsub(pattern = "B cell",replacement = "B cell activated") %>%

gsub(pattern = "Monocyte",replacement = "Monocytes") %>%

gsub(pattern = "Neutrophil",replacement = "Neutrophils")

pData(GSE49910)$batch = "GSE49910"

use <- grep(pattern = "(_)|(Erythroblast)|(Bone marrow)",pData(GSE49910)$title,invert = T)

GSE49910<- GSE49910[,use]

head(pData(GSE49910))

colnames(pData(GSE49910))[1] = "cell.subtype"

GSE49910 = anno.GEO(GEO = GSE49910)

exprs(GSE49910) = log2(exprs(GSE49910))

# GSE49910 = GSE49910[,1:37]

saveRDS(GSE49910,file = file.path(runtime.path,"GSE49910.rds"))

}else(

GSE49910 = readRDS(file.path(runtime.path,"GSE49910.rds"))

)

#3 Th17 cells

if(!file.exists(file.path(runtime.path,"GSE51540.rds"))){

GSE51540 = getGEO("GSE51540", GSEMatrix =TRUE, AnnotGPL=TRUE,destdir = file.path(runtime.path))

if (length(GSE51540) > 1) idx <- grep("GPL16268", attr(GSE51540, "names")) else idx <- 1

GSE51540<- GSE51540[[idx]]

pData(GSE51540) = pData(GSE51540)[,1:2]

con = grep(pattern = "Control",pData(GSE51540)$title)

GSE51540 = GSE51540[,con]

pData(GSE51540)$title = "T helper 17"

pData(GSE51540)$batch = "GSE51540"

colnames(pData(GSE51540))[1] = "cell.subtype"

GSE51540 = anno.GEO2(GEO = GSE51540)

saveRDS(GSE51540,file = file.path(runtime.path,"GSE51540.rds"))

}else(

GSE51540 = readRDS(file.path(runtime.path,"GSE51540.rds"))

)

#4 DC.cells

if(!file.exists(file.path(runtime.path,"GSE59237.rds"))){

GSE59237 = getGEO("GSE59237", GSEMatrix =TRUE, AnnotGPL=TRUE,destdir = file.path(runtime.path))

if (length(GSE59237) > 1) idx <- grep("GPL570", attr(GSE59237, "names")) else idx <- 1

GSE59237<- GSE59237[[idx]]

pData(GSE59237) = pData(GSE59237)[,1:2]

# con = grep(pattern = "Control",pData(GSE59237)$title)

GSE59237 = GSE59237[,1:10]

pData(GSE59237)$title = c(rep("Dendritic cells resting",6),rep("Dendritic cells activated",4))

pData(GSE59237)$batch = "GSE59237"

head(pData(GSE59237))

colnames(pData(GSE59237))[1] = "cell.subtype"

GSE59237 = anno.GEO(GEO = GSE59237)

exprs(GSE59237) = log2(exprs(GSE59237))

saveRDS(GSE59237,file = file.path(runtime.path,"GSE59237.rds"))

}else(

GSE59237 = readRDS(file.path(runtime.path,"GSE59237.rds"))

)

#5 iDCs

if(!file.exists(file.path(runtime.path,"GSE6863.rds"))){

GSE6863 = getGEO("GSE6863", GSEMatrix =TRUE, AnnotGPL=TRUE,destdir = file.path(runtime.path))

if (length(GSE6863) > 1) idx <- grep("GPL570", attr(GSE6863, "names")) else idx <- 1

GSE6863<- GSE6863[[idx]]

pData(GSE6863) = pData(GSE6863)[,1:2]

GSE6863 = GSE6863[,4:6]

pData(GSE6863)$title = "Immature dendritic cells"

pData(GSE6863)$batch = "GSE6863"

head(pData(GSE6863))

colnames(pData(GSE6863))[1] = "cell.subtype"

GSE6863 = anno.GEO(GEO = GSE6863)

exprs(GSE6863) = log2(exprs(GSE6863))

saveRDS(GSE6863,file = file.path(runtime.path,"GSE6863.rds"))

}else(

GSE6863 = readRDS(file.path(runtime.path,"GSE6863.rds"))

)

#6 NK CD8.Tcells 待修改

if(!file.exists(file.path(runtime.path,"GSE8059.rds"))){

GSE8059 = getGEO("GSE8059", GSEMatrix =TRUE, AnnotGPL=TRUE,destdir = file.path(runtime.path))

if (length(GSE8059) > 1) idx <- grep("GPL570", attr(GSE8059, "names")) else idx <- 1

GSE8059<- GSE8059[[idx]]

GSE8059<- GSE8059[,c(1:4)]

pData(GSE8059) = pData(GSE8059)[,1:2]

pData(GSE8059)$batch = "GSE8059"

pData(GSE8059)$title = c("NK resting",rep("NK activated"))

colnames(pData(GSE8059))[1] = "cell.subtype"

GSE8059 = anno.GEO(GEO = GSE8059)

exprs(GSE8059) = log2(exprs(GSE8059))

saveRDS(GSE8059,file = file.path(runtime.path,"GSE8059.rds"))

}else(

GSE8059 = readRDS(file.path(runtime.path,"GSE8059.rds"))

)

#7 gamma_delta.Tcells normal.lymphocyte

if(!file.exists(file.path(runtime.path,"GSE13906.rds"))){

GSE13906 = getGEO("GSE13906", GSEMatrix =TRUE, AnnotGPL=TRUE,destdir = file.path(runtime.path))

if (length(GSE13906) > 1) idx <- grep("GPL570", attr(GSE13906, "names")) else idx <- 1

GSE13906<- GSE13906[[idx]]

GSE13906<- GSE13906[,c(1:2)]

pData(GSE13906) = pData(GSE13906)[,1:2]

pData(GSE13906)$title = c("T gamma delta","T gamma delta")

pData(GSE13906)$batch = "GSE13906"

head(pData(GSE13906))

colnames(pData(GSE13906))[1] = "cell.subtype"

GSE13906 = anno.GEO(GEO = GSE13906)

exprs(GSE13906) = log2(exprs(GSE13906))

GSE13906 = GSE13906[,1:2]

saveRDS(GSE13906,file = file.path(runtime.path,"GSE13906.rds"))

}else(

GSE13906 = readRDS(file.path(runtime.path,"GSE13906.rds"))

)

#8 DCs derived from CD14+ monocytes待

if(!file.exists(file.path(runtime.path,"GSE23371.rds"))){

GSE23371 = getGEO("GSE23371", GSEMatrix =TRUE, AnnotGPL=TRUE,destdir = file.path(runtime.path))

if (length(GSE23371) > 1) idx <- grep("GPL570", attr(GSE23371, "names")) else idx <- 1

GSE23371<- GSE23371[[idx]]

GSE23371<- GSE23371[,1:3]

pData(GSE23371) = pData(GSE23371)[,1:2]

pData(GSE23371)$batch = "GSE23371"

pData(GSE23371)$title = "Immature dendritic cells"

colnames(pData(GSE23371))[1] = "cell.subtype"

GSE23371 = anno.GEO(GEO = GSE23371)

exprs(GSE23371) = log2( exprs(GSE23371)) # negitive value

saveRDS(GSE23371,file = file.path(runtime.path,"GSE23371.rds"))

}else(

GSE23371 = readRDS(file.path(runtime.path,"GSE23371.rds"))

)

#9 HMC-1 cell 人肥大细胞

if(!file.exists(file.path(runtime.path,"GSE25320.rds"))){

GSE25320 = getGEO("GSE25320", GSEMatrix =TRUE, AnnotGPL=TRUE,destdir = file.path(runtime.path))

if (length(GSE25320) > 1) idx <- grep("GPL570", attr(GSE25320, "names")) else idx <- 1

GSE25320<- GSE25320[[idx]]

GSE25320<- GSE25320[,5:8]

pData(GSE25320) = pData(GSE25320)[,1:2]

pData(GSE25320)$batch = "GSE25320"

pData(GSE25320)$title = "Mast cells activated"

colnames(pData(GSE25320))[1] = "cell.subtype"

GSE25320 = anno.GEO(GEO = GSE25320)

exprs(GSE25320) = log2( exprs(GSE25320)) # negitive value

saveRDS(GSE25320,file = file.path(runtime.path,"GSE25320.rds"))

c}else(

GSE25320 = readRDS(file.path(runtime.path,"GSE25320.rds"))

)

#10 gamma_delta.cells

if(!file.exists(file.path(runtime.path,"GSE27291.rds"))){

GSE27291 = getGEO("GSE27291", GSEMatrix =TRUE, AnnotGPL=TRUE,destdir = file.path(runtime.path))

if (length(GSE27291) > 1) idx <- grep("GPL570", attr(GSE27291, "names")) else idx <- 1

GSE27291<- GSE27291[[idx]]

con = grep(pattern = "IL2",x = pData(GSE27291)$characteristics_ch1.2)

GSE27291<- GSE27291[,con]

pData(GSE27291) = pData(GSE27291)[,1:2]

pData(GSE27291)$title = "T gamma delta"

pData(GSE27291)$batch = "GSE27291"

colnames(pData(GSE27291))[1] = "cell.subtype"

GSE27291 = anno.GEO(GEO = GSE27291)

exprs(GSE27291) = log2(exprs(GSE27291))

saveRDS(GSE27291,file = file.path(runtime.path,"GSE27291.rds"))

c}else(

GSE27291 = readRDS(file.path(runtime.path,"GSE27291.rds"))

)

#11 NK.cells

if(!file.exists(file.path(runtime.path,"GSE27838.rds"))){

GSE27838 = getGEO("GSE27838", GSEMatrix =TRUE, AnnotGPL=TRUE,destdir = file.path(runtime.path))

if (length(GSE27838) > 1) idx <- grep("GPL570", attr(GSE27838, "names")) else idx <- 1

GSE27838<- GSE27838[[idx]]

con = grep(pattern = "^NK_Donor",pData(GSE27838)$title)

GSE27838<- GSE27838[,con]

pData(GSE27838) = pData(GSE27838)[,1:2]

pData(GSE27838)$title = "NK activated"

pData(GSE27838)$batch = "GSE27838"

# GSE27838$title %<>% as.character() %>% gsub(pattern = "_Donor_\\d{2}",replacement = ".cell")

head(pData(GSE27838))

colnames(pData(GSE27838))[1] = "cell.subtype"

GSE27838 = anno.GEO(GEO = GSE27838)

exprs(GSE27838) = log2(exprs(GSE27838))

saveRDS(GSE27838,file = file.path(runtime.path,"GSE27838.rds"))

}else(

GSE27838 = readRDS(file.path(runtime.path,"GSE27838.rds"))

)

#12 Bcell CD4_Tcell CD8_Tcell mono_DC.cell plasma_DC.cell

if(!file.exists(file.path(runtime.path,"GSE28490.rds"))){

GSE28490 = getGEO("GSE28490", GSEMatrix =TRUE, AnnotGPL=TRUE,destdir = file.path(runtime.path))

if (length(GSE28490) > 1) idx <- grep("GPL570", attr(GSE28490, "names")) else idx <- 1

GSE28490<- GSE28490[[idx]]

pData(GSE28490) = pData(GSE28490)[,1:2]

GSE28490<- GSE28490[,11:15]

# pData(GSE28490)$title %<>% as.character() %>% gsub(pattern = " rep\\d{1,2} mRNA \\(Roche\\)",replacement = "") %>%

# gsub(pattern = "B cells",replacement = "Bcell") %>%

# gsub(pattern = "CD4\\+ T cells",replacement ="CD4.Tcell" ) %>%

# gsub(pattern = "CD8\\+ T cells",replacement ="CD8.Tcell" ) %>%

# gsub(pattern = "mDC",replacement ="Immature.DC.cell" ) %>%

# gsub(pattern = "pDC",replacement ="plas.DC.cell" ) %>%

# gsub(pattern = "NK cells",replacement ="NK.cell") %>%

# gsub(pattern = "Monocytes",replacement ="Monocyte") %>%

# gsub(pattern = "Neutrophils",replacement ="Neutrophil")

#

pData(GSE28490)$title = "B cell activated"

pData(GSE28490)$batch = "GSE28490"

head(pData(GSE28490))

colnames(pData(GSE28490))[1] = "cell.subtype"

GSE28490 = anno.GEO(GEO = GSE28490)

saveRDS(GSE28490,file = file.path(runtime.path,"GSE28490.rds"))

}else(

GSE28490 = readRDS(file.path(runtime.path,"GSE28490.rds"))

)

#13 HSCs

if(!file.exists(file.path(runtime.path,"GSE28698.rds"))){

GSE28698 = getGEO("GSE28698", GSEMatrix =TRUE, AnnotGPL=TRUE,destdir = file.path(runtime.path))

if (length(GSE28698) > 1) idx <- grep("GPL570", attr(GSE28698, "names")) else idx <- 1

GSE28698<- GSE28698[[idx]]

GSE28698<- GSE28698[,c(1,3,5)]

pData(GSE28698) = pData(GSE28698)[,1:2]

pData(GSE28698)$batch = "GSE28698"

pData(GSE28698)$title = "Eosinophils"

colnames(pData(GSE28698))[1] = "cell.subtype"

GSE28698 = anno.GEO(GEO = GSE28698)

exprs(GSE28698) = log2(exprs(GSE28698))

saveRDS(GSE28698,file = file.path(runtime.path,"GSE28698.rds"))

}else(

GSE28698 = readRDS(file.path(runtime.path,"GSE28698.rds"))

)

#14 CD4.Tcell NK.cell Va24.Tcell

if(!file.exists(file.path(runtime.path,"GSE28726.rds"))){

GSE28726 = getGEO("GSE28726", GSEMatrix =TRUE, AnnotGPL=TRUE,destdir = file.path(runtime.path))

if (length(GSE28726) > 1) idx <- grep("GPL570", attr(GSE28726, "names")) else idx <- 1

GSE28726<- GSE28726[[idx]]

con.CD4.resting = pData(GSE28726)$title %>% as.character() %>% grep(pattern = "CD4-\\d{1,2}-N$")

con.CD4.activated = pData(GSE28726)$title %>% as.character() %>% grep(pattern = "CD4-\\d{1,2}-S$")

con.NKT = pData(GSE28726)$title %>% as.character() %>% grep(pattern = "NKT-\\d{1,2}-S")

GSE28726<- GSE28726[,c(con.CD4.activated,con.CD4.resting,con.NKT)]

pData(GSE28726) = pData(GSE28726)[,c(1,2)]

pData(GSE28726)$title = c(rep("CD4 T cell activated",4),rep("CD4 T cell resting",4),rep("NKT activated",6))

pData(GSE28726)$batch = "GSE28726"

head(pData(GSE28726))

colnames(pData(GSE28726))[1] = "cell.subtype"

GSE28726 = anno.GEO(GEO = GSE28726)

exprs(GSE28726)= log2(exprs(GSE28726))

saveRDS(GSE28726,file = file.path(runtime.path,"GSE28726.rds"))

}else(

GSE28726 = readRDS(file.path(runtime.path,"GSE28726.rds"))

)

#15 plasma_DC.cell

if(!file.exists(file.path(runtime.path,"GSE37750.rds"))){

GSE37750 = getGEO("GSE37750", GSEMatrix =TRUE, AnnotGPL=TRUE,destdir = file.path(runtime.path))

if (length(GSE37750) > 1) idx <- grep("GPL570", attr(GSE37750, "names")) else idx <- 1

GSE37750<- GSE37750[[idx]]

GSE37750<- GSE37750[,1:8]

pData(GSE37750) = pData(GSE37750)[,c(1,2)]

pData(GSE37750)$title = "Plasmacytoid dendritic cells"

pData(GSE37750)$batch = "GSE37750"

head(pData(GSE37750))

colnames(pData(GSE37750))[1] = "cell.subtype"

GSE37750 = anno.GEO(GEO = GSE37750)

saveRDS(GSE37750,file = file.path(runtime.path,"GSE37750.rds"))

}else(

GSE37750 = readRDS(file.path(runtime.path,"GSE37750.rds"))

)

#16 neutrophils

if(!file.exists(file.path(runtime.path,"GSE39889.rds"))){

GSE39889 = getGEO("GSE39889", GSEMatrix =TRUE, AnnotGPL=TRUE,destdir = file.path(runtime.path))

if (length(GSE39889) > 1) idx <- grep("GPL570", attr(GSE39889, "names")) else idx <- 1

GSE39889<- GSE39889[[idx]]

pData(GSE39889) = pData(GSE39889)[,c(8,2)]

con = grep(pattern = "control",pData(GSE39889)$source_name_ch1)

GSE39889<- GSE39889[,con]

pData(GSE39889)$batch = "GSE39889"

pData(GSE39889)$source_name_ch1 = "Neutrophils"

head(pData(GSE39889))

colnames(pData(GSE39889))[1] = "cell.subtype"

GSE39889 = anno.GEO(GEO = GSE39889)

exprs(GSE39889) = log2(exprs(GSE39889))

saveRDS(GSE39889,file = file.path(runtime.path,"GSE39889.rds"))

}else(

GSE39889 = readRDS(file.path(runtime.path,"GSE39889.rds"))

)

#### --------------------- TCGA ---------------------

TCGA.exp = fread("data/TCGA-KIRC/TCGA-KIRC.htseq_counts.tsv.gz",data.table = F)

rownames(TCGA.exp) = TCGA.exp$Ensembl_ID

TCGA.exp$Ensembl_ID = NULL

dim(TCGA.exp) #60483 607

TCGA.survival = fread("data/TCGA-KIRC/TCGA-KIRC.survival.tsv.gz",data.table = F)

TCGA.survival = TCGA.survival[!duplicated(TCGA.survival$`_PATIENT`),]

dim(TCGA.survival) #533 4

head(TCGA.survival)

TCGA.pheno = fread("data/TCGA-KIRC/TCGA-KIRC.GDC_phenotype.tsv.gz",data.table = F) %>%

subset(.,sample_type.samples!="Solid Tissue Normal")

head(TCGA.pheno)

dim(TCGA.pheno)

both.sample = intersect(intersect(colnames(TCGA.exp),TCGA.survival$sample),TCGA.pheno$submitter_id.samples)

length(both.sample) #307

TCGA.survival = subset(TCGA.survival,sample%in%both.sample) %$% .[order(sample),]

TCGA.exp = TCGA.exp[,both.sample]

TCGA.exp = TCGA.exp[,order(colnames(TCGA.exp))]

head(TCGA.pheno)

TCGA.pheno = subset(TCGA.pheno,submitter_id.samples%in%both.sample)

data.table::fwrite(TCGA.exp,file = file.path(result.path,"TCGA.exp.tsv"),quote = F,row.names = T,sep = "\t")

data.table::fwrite(TCGA.survival,file = file.path(result.path,"TCGA.survival.tsv"),quote = F,row.names = F,sep = "\t")

data.table::fwrite(TCGA.pheno,file = file.path(result.path,"TCGA.pheno.tsv"),quote = F,row.names = F,sep = "\t")

#### --------------------- GEO GSE22541 ---------------------

gset <- getGEO("GSE22541", GSEMatrix =TRUE, AnnotGPL=FALSE,destdir = file.path(runtime.path))

if (length(gset) > 1) idx <- grep("GPL570", attr(gset, "names")) else idx <- 1

GSE22541 <- gset[[idx]]

a = pData(GSE22541)[1:20,c(2,10,11)]

a$characteristics_ch1 = ifelse(a$characteristics_ch1=="gender: m","male","female")

a$characteristics_ch1.1 %<>% gsub(pattern = "Disease free interval \\(DFI\\) in months: ",replacement ="")

a$DFI = "1"

a = a[,c(1,2,4,3)]

colnames(a) = c("geo_accession","gender","DFI","DFI.time")

b = pData(GSE22541)[21:44,c(2,11,16)]

b$characteristics_ch1.1 %<>% as.character() %>% gsub(pattern = "gender: ",replacement ="")

b$PFI = ifelse(grepl(pattern = "Follow-up",as.character(b$characteristics_ch1.6)),"0","1")

b$characteristics_ch1.6 = as.character(b$characteristics_ch1.6) %>% gsub(pattern = "(dfs/follow-up: )",replacement = "") %>% gsub(pattern = ".* = (\\d*) month[s]?",replacement = "\\1")

b = b[,c(1,2,4,3)]

colnames(b) = c("geo_accession","gender","DFI","DFI.time")

c = pData(GSE22541)[45:68,c(2,10,12)]

c$characteristics_ch1 %<>% as.character() %>% gsub(pattern = "gender: ",replacement ="")

c$characteristics_ch1.2 = as.character(c$characteristics_ch1.2) %>% gsub(pattern = "(dfs/follow-up: )",replacement = "") %>% gsub(pattern = ".* = (\\d*) month[s]?",replacement = "\\1")

c$PFI = "1"

c = c[,c(1,2,4,3)]

colnames(c) = c("geo_accession","gender","DFI","DFI.time")

pData(GSE22541) = purrr::reduce(list(a,b,c),rbind)

# pData(GSE22541) = purrr::reduce(list(b),rbind)

GSE22541 = anno.GEO(GEO = GSE22541)

# GSE22541 = GSE22541[,row.names(pData(GSE22541))]

# exprs(GSE22541)= log2(exprs(GSE22541))

exprs(GSE22541)= log2(exprs(GSE22541))

saveRDS(GSE22541,file = file.path(runtime.path,"GSE22541.rds"))

#### --------------------- GSE29609 --------------------------

gset <- getGEO("GSE29609", GSEMatrix =TRUE, AnnotGPL=FALSE,destdir = file.path(runtime.path))

if (length(gset) > 1) idx <- grep("GPL1708", attr(gset, "names")) else idx <- 1

GSE29609 <- gset[[idx]]

subset(fData(GSE29609),GENE=="84689")

head(pData(GSE29609))

colnames(pData(GSE29609))

a = pData(GSE29609)[,c(2,10:13,59,72)]

a$characteristics_ch1 %<>% as.character() %>% gsub(pattern = "age at diagnosis \\(y\\): ",replacement = "")

a$characteristics_ch1.1 %<>% as.character() %>% gsub(pattern = "t \\(tnm stage\\): ",replacement ="")

a$characteristics_ch1.2 %<>% as.character() %>% gsub(pattern = "n \\(tnm stage\\): ",replacement ="")

a$characteristics_ch1.3 %<>% as.character() %>% gsub(pattern = "m \\(tnm stage\\): ",replacement ="")

colnames(a) = c("geo_accession","age","t","n","m","OS","OS.time")

head(a)

pData(GSE29609) = a

GSE29609 = anno.GEO(GEO = GSE29609)

saveRDS(GSE29609,file = file.path(runtime.path,"GSE29609.rds"))

#### --------------------- KIRC cancer cell line ---------------------

# if(!file.exists(file.path(runtime.path,"GSE2454.rds"))){

# GSE2454 = getGEO("GSE2454", GSEMatrix =TRUE, AnnotGPL=TRUE,destdir = file.path(runtime.path))

# if (length(GSE2454 ) > 1) idx <- grep("GPL1530", attr(GSE2454 , "names")) else idx <- 1

# GSE2454 <- GSE2454 [[idx]]

# pData(GSE2454 ) = pData(GSE2454 )[,c(8,2)]

#

# pData(GSE2454 )$batch = "GSE2454"

# # pData(GSE2454 )$source_name_ch1 = "Neutrophil"

# head(pData(GSE2454 ))

# colnames(pData(GSE2454 ))[1] = "cell.subtype"

# pData(GSE2454 )[1] = "KIRC.cell.line"

# GSE2454 = anno.GEO(GEO = GSE2454 )

# # exprs(GSE2454 ) = log2(exprs(GSE2454 ))

# saveRDS(GSE2454 ,file = file.path(runtime.path,"GSE2454.rds"))

# }else(

# GSE2454 = readRDS(file.path(runtime.path,"GSE2454.rds"))

# )

# GSE81812 = fread("v1/runtime/01.原始数据/GSE81812_Normalized_counts.txt.gz")

# GSE81812. = data.frame(row.names = GSE81812$V1,GSE81812[,-1])

# head(GSE81812.)

# dim(GSE81812.)

#

# colnames(GSE81812.) %<>% gsub(pattern = "(Unkonwn.180.)|(.bam)|(merge_Sample_Pan_[BC].)|(_mapping.bam)|(Sample_Pan_[BC].)",replacement = "")

# GSE81812 = GSE81812.

# GSE81812[1:5,1:15]

# colnames(GSE81812)

#

#

# GSE81812.celltype = readLines("v1/runtime/01.原始数据/GSE81812_series_matrix.txt.gz")[c(29:30)] %>% fread(text = .,header = F) %>% '['(,-1)

# dim(GSE81812.celltype)

# GSE81812.celltype[,1:6]

# head(GSE81812.celltype,20)

#

#

# GSE81812.celltype.KIRC = subset(GSE81812.celltype,`Cancer Type\r\n(matching TCGA label)`=="KIRC")

#

# use.col.GSE81812 = intersect(colnames(GSE81812),GSE81812.celltype.KIRC$`Sample Name`)

# GSE81812.KIRC = GSE81812[,use.col.GSE81812]

#

# GSE81812.KIRC. = apply(GSE81812.KIRC,2,as.numeric)

# rownames(GSE81812.KIRC.) = rownames(GSE81812.KIRC)

# GSE81812.KIRC = GSE81812.KIRC.[complete.cases(GSE81812.KIRC.),]

# # GSE81812.KIRC[1:6,1:3]

#

# GSE81812 = ExpressionSet(assayData = as.matrix(log2(GSE81812.KIRC+1))) # 34985 35

# pData(GSE81812) = data.frame(cell.subtype="KIRC cell line",ID=colnames(GSE81812.KIRC),batch="GSE81812",row.names = colnames(GSE81812.KIRC))

# saveRDS(GSE81812,file = file.path(runtime.path,"GSE81812.rds"))

#

#

#

# if(!file.exists(file.path(runtime.path,"GSE23806.rds"))){

# GSE23806 = getGEO("GSE23806", GSEMatrix =TRUE, AnnotGPL=TRUE,destdir = file.path(runtime.path))

# if (length(GSE23806 ) > 1) idx <- grep("GPL570", attr(GSE23806 , "names")) else idx <- 1

# GSE23806 <- GSE23806 [[idx]]

# pData(GSE23806 ) = pData(GSE23806 )[,c(37,2)]

#

# pData(GSE23806 )$batch = "GSE23806"

# # pData(GSE23806 )$source_name_ch1 = "Neutrophil"

# head(pData(GSE23806 ))

# colnames(pData(GSE23806 ))[1] = "cell.subtype"

# pData(GSE23806)[1] = "KIRC.cell.line"

# GSE23806 = anno.GEO(GEO = GSE23806 )

# # exprs(GSE23806 ) = log2(exprs(GSE23806 ))

# saveRDS(GSE23806 ,file = file.path(runtime.path,"GSE23806.rds"))

# }else(

# GSE23806 = readRDS(file.path(runtime.path,"GSE23806.rds"))

# )

#### --------------------- CCLE ---------------------

CCLE = fread("data/KIRC-Cell line/CCLE/CCLE_RNAseq_rsem_genes_tpm_20180929.txt.gz")

CCLE.celltype = fread("data/KIRC-Cell line/CCLE/Cell_lines_annotations_20181226.txt")

CCLE.celltype.KIRC = CCLE.celltype[tcga_code=="KIRC"]

dim(CCLE.celltype.KIRC)

CCLE.KIRC.cell.line = intersect(colnames(CCLE),CCLE.celltype.KIRC$CCLE_ID)

use.col = c("gene_id",CCLE.KIRC.cell.line)

CCLE.KIRC = CCLE[,..use.col]

CCLE.KIRC$gene_id %<>% gsub(pattern = "(ENSG\\d{11})\\.\\d{1,2}",replacement = "\\1")

CCLE.KIRC$gene_id = mapIds(org.Hs.eg.db,keys = CCLE.KIRC$gene_id,column = "SYMBOL",keytype = "ENSEMBL")

CCLE.KIRC = CCLE.KIRC[!is.na(gene_id),]

CCLE.KIRC = as.data.frame(CCLE.KIRC)

CCLE.KIRC = dup.genes(CCLE.KIRC)

CCLE = ExpressionSet(assayData = as.matrix(log2(CCLE.KIRC+1))) # 20530 515

pData(CCLE) = data.frame(cell.subtype="KIRC cell line",ID=colnames(CCLE.KIRC),batch="CCLE",row.names = colnames(CCLE.KIRC))

saveRDS(CCLE,file = file.path(runtime.path,"CCLE.rds"))

#### --------------------- GDSC ---------------------

GDSC = fread("data/KIRC-Cell line/GDSC/rnaseq_fpkm_20191101.csv")

GDSC= GDSC[-c(1,3,4),-1]

GDSC. = data.frame(row.names = GDSC$V2[-1],GDSC[-1,-1])

colnames(GDSC.) = as.character(GDSC[1,-1])

GDSC = GDSC.

GDSC[1:5,1:15]

GDSC.celltype = readxl::read_xlsx("data/KIRC-Cell line/GDSC/Cell_Lines_Details.xlsx")

GDSC.celltype.KIRC = subset(GDSC.celltype,`Cancer Type\r\n(matching TCGA label)`=="KIRC")

use.col.GDSC = intersect(colnames(GDSC),GDSC.celltype.KIRC$`Sample Name`)

GDSC.KIRC = GDSC[,use.col.GDSC]

GDSC.KIRC. = apply(GDSC.KIRC,2,as.numeric)

rownames(GDSC.KIRC.) = rownames(GDSC.KIRC)

GDSC.KIRC = GDSC.KIRC.[complete.cases(GDSC.KIRC.),]

# GDSC.KIRC[1:6,1:3]

GDSC = ExpressionSet(assayData = as.matrix(log2(GDSC.KIRC+1))) # 34985 35

pData(GDSC) = data.frame(cell.subtype="KIRC cell line",ID=colnames(GDSC.KIRC),batch="GDSC",row.names = colnames(GDSC.KIRC))

saveRDS(GDSC,file = file.path(runtime.path,"GDSC.rds"))

#### --------------------- 免疫治疗数据 ---------------------

# GSE79671.ex = fread("v1/runtime/01.原始数据/GSE79671_CountMatrix.txt.gz")

# head(GSE79671.ex)

# GSE79671.cli = readLines("v1/runtime/01.原始数据/GSE79671_series_matrix.txt.gz")

# GSE79671.cli[c(28,35,39,41,42,43,47)]

save.image(file = "v1/01.原始数据.rds",compress = "gzip")
