# Supplementary material for: Significance of CD8+ T cell infiltration-related biomarkers and the corresponding prediction model for the prognosis of kidney renal clear cell carcinoma
Source: Aging (Albany NY). 2021 Oct 4;13(19):22912–33. doi: 10.18632/aging.203584 (PMC8544304; doi:10.18632/aging.203584)
Supplement: Supplementary Figures [file aging-13-203584-s005.pdf]

## SUPPLEMENTARY FIGURES

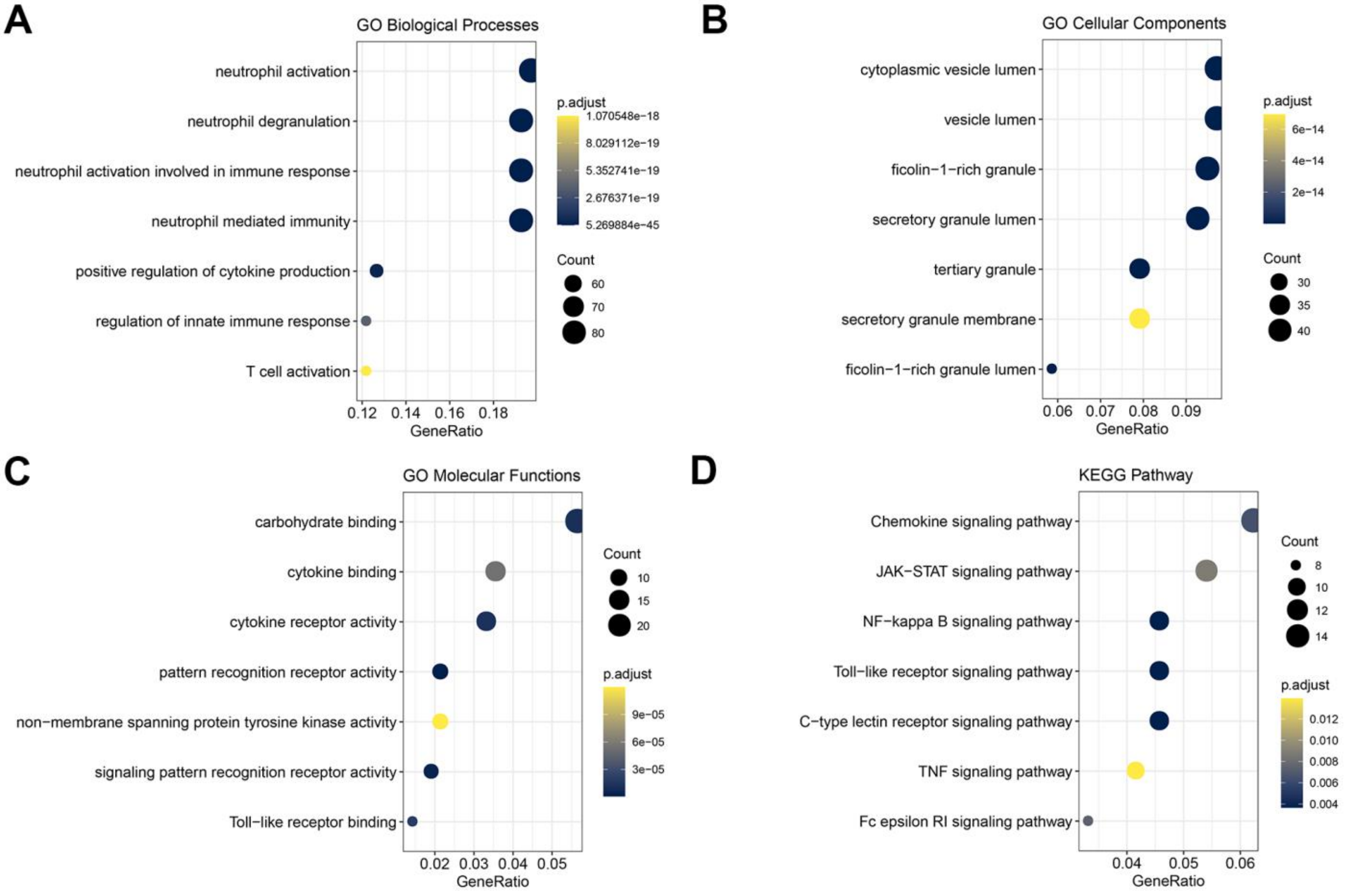


**Supplementary Figure 1. Enrichment analysis of gene ontology (GO) and kyoto encyclopedia of genes and genomes (KEGG) specific marker genes for TIL-CD8 T cells.** The size of the dot indicates the number of genes enriched in the items; The color of the dot indicates the size of the p-value after correction. (**A**) Biological process of GO enrichment analysis: items such as neutrophil activation, neutrophil degranulation, neutrophil activation involved in immune response, neutrophil mediated immunity, positive regulation of cytokine production, regulation of innate immune response and T cell activation were mainly included in the biological process. (**B**) Molecular functions of GO enrichment analysis: items such as carbohydrate binding, cytokine binding, cytokine receptor activity, pattern recognition receptor activity, non-membrane spanning protein tyrosine kinase activity, signaling pattern recognition receptor activity and Toll-like receptor binding were mainly included in the analysis. (**C**) Cellular component of GO enrichment analysis: items such as cytoplasmic vesicle lumen, vesicle lumen, ficolin−1−rich granule, secretory granule lumen, tertiary granule, secretory granule membrane and ficolin−1−rich granule lumen were mainly included in the analysis. (**D**) KEGG pathway enrichment analysis of marker genes showed that the genes were mainly enriched in Chemokine signaling pathway, JAK-STAT signaling pathway, NF-kappa B signaling pathway, Toll-like receptor signaling pathway, C-type lectin receptor signaling pathway, and TNF signaling pathway and Fcepsilon RI signaling pathway.

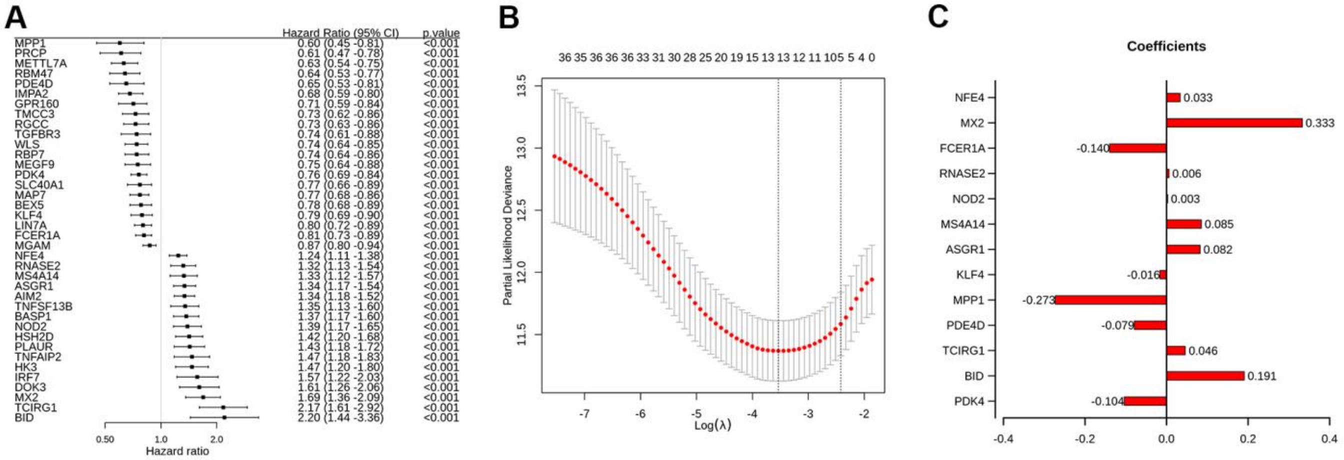


**Supplementary Figure 2. Univariate Cox and LASSO regression analysis.** 38 TIL-CD8T-related genes related to the prognosis of KIRC were screened by univariate Cox regression analysis, and then LASSO regression analysis was used to reduce dimensionality, and 13 genes related to the prognosis of KIRC were obtained. (**A**) Forest plot of univariate Cox regression analysis. (**B**) Coefficient distribution of LASSO regression analysis. (**C**) Regression equation coefficients of 13 prognostic-related factors screened by LASSO regression analysis.

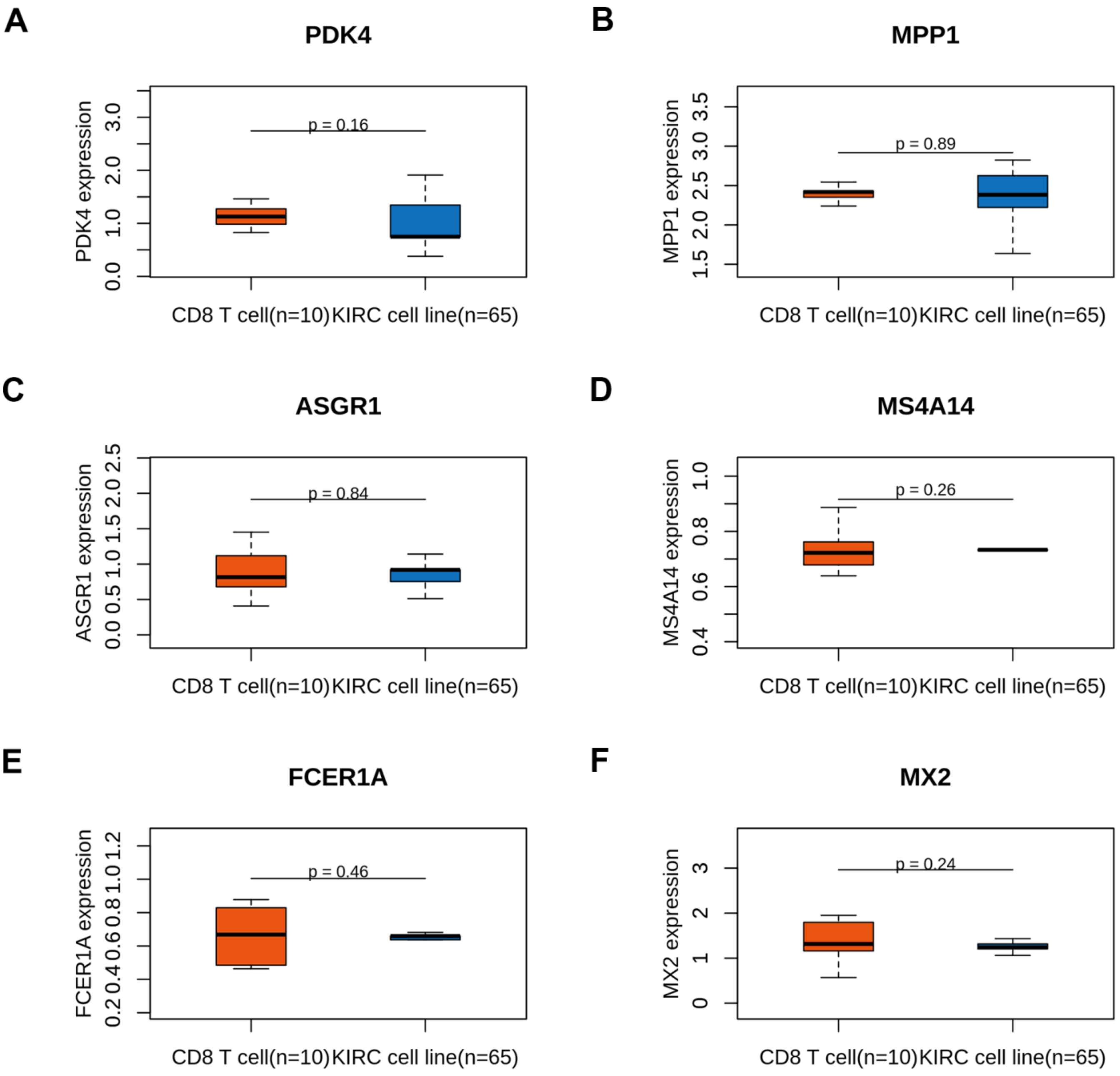


**Supplementary Figure 3. The expression status of prognostic factors contained in the model in CD8 T cells and KIRC cell lines.** The significance of the differential expression in CD8 T cell line and KIRC cell line was analyzed by Wilcoxon rank sum test. The abscissa axis represents CD8 T cell line and KIRC cell line; The ordinate axis represents the expression level of related genes. (**A**) The differential expression of the prognostic factor PDK4 between CD8 T cells and KIRC cell lines. (**B**) The differential expression of the prognostic factor MPP1 between CD8 T cells and KIRC cell lines. (**C**) The differential expression of the prognostic factor ASGR1 between CD8 T cells and KIRC cell lines. (**D**) The differential expression of the prognostic factor MS4A14 between CD8 T cells and KIRC cell lines. (**E**) The differential expression of the prognostic factor FCER1A between CD8 T cells and KIRC cell lines. (**F**) The differential expression of the prognostic factor MX2 between CD8 T cells and KIRC cell lines.

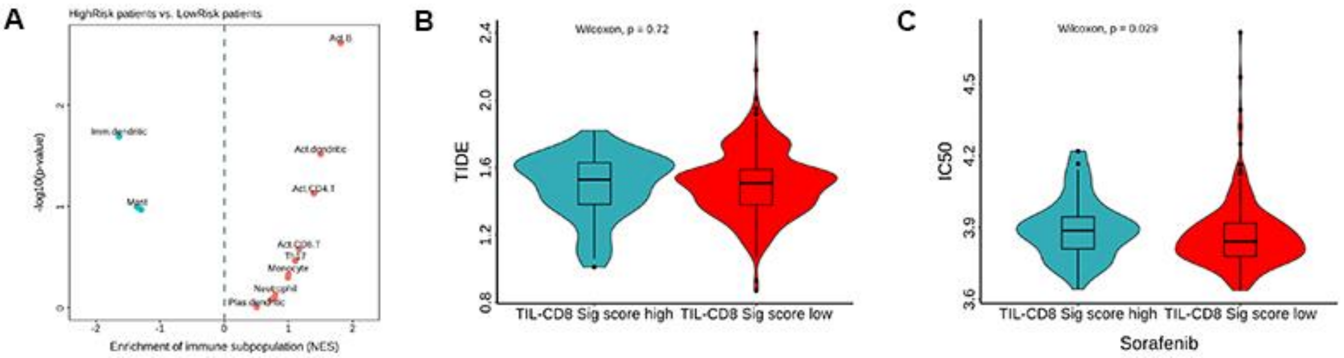


**Supplementary Figure 4.** (**A**) Volcano map enriched with NES scores of tumor immune cell subgroup GSEA scored by TIL-CD8Sig: The single-sample GSEA analysis was used to evaluate the level of immune cell infiltration in the TIL-CD8T Sig score groups. (**B**) TIDE score results of different TIL-CD8Sig score groups. The calculation of the tumor immune dysfunction and exclusion (TIDE) score was finished by the TIDE online analysis website (http://tide.dfci.harvard.edu/). The abscissa axis represents different TIL-CD8Sig score groups; the ordinate axis represents the TIDE score. (**C**) The prediction result of sorafenib resistance in different TIL-CD8 Sig score subgroups. The prediction of sorafenib drug sensitivity was completed by the R software package pRRophetic.

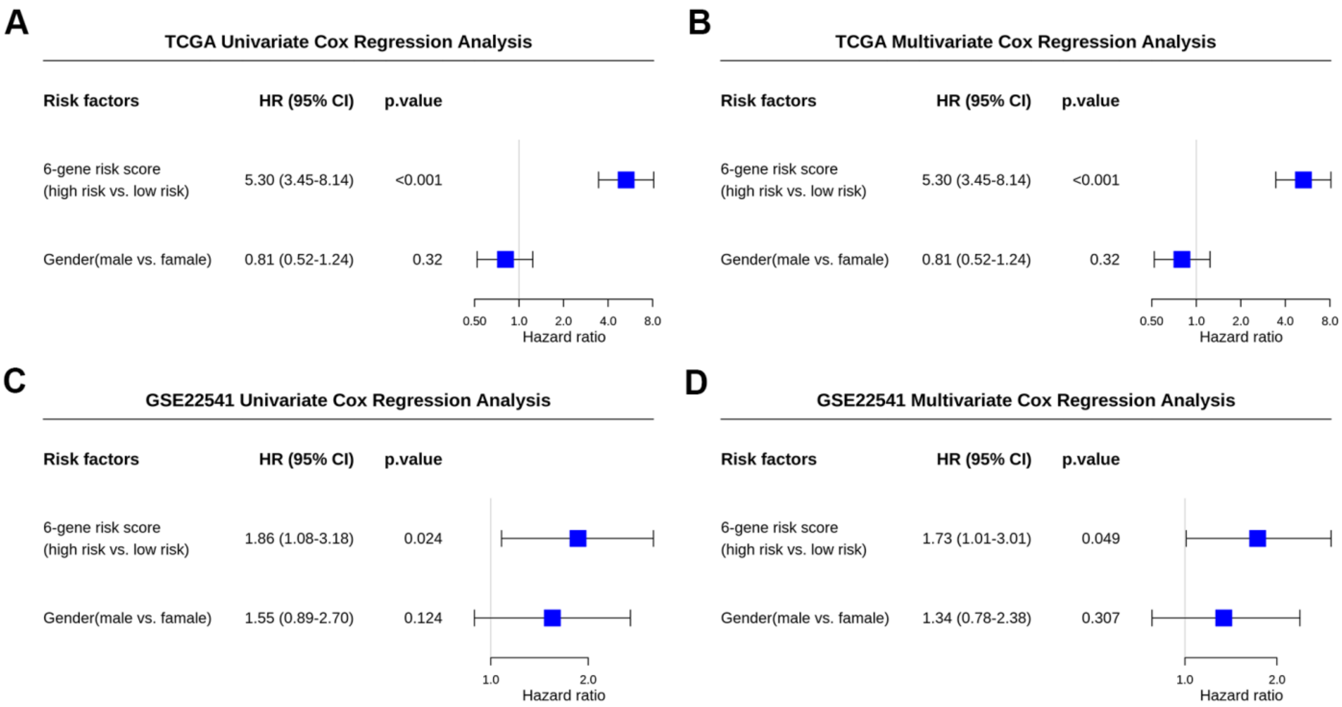


**Supplementary Figure 5. Verification results on the independent prediction efficiency of TIL-CD8Sig score.** Univariate Cox regression and multivariate Cox regression analysis were used to verify the data of TCGA-KIRC and GSE22541 in KIRC patients. (**A**) The Forest plot of TCGA-KIRC Univariate Cox Regression Analysis. (**B**) The Forest plot of TCGA-KIRC Multivariate Cox Regression Analysis. (**C**) The Forest plot of GSE22541 Univariate Cox Regression Analysis. (**D**) The Forest plot of GSE22541 Multivariate Cox Regression Analysis.
