# Supplementary material for: Significance of CD8+ T cell infiltration-related biomarkers and the corresponding prediction model for the prognosis of kidney renal clear cell carcinoma
Source: Aging (Albany NY). 2021 Oct 4;13(19):22912–33. doi: 10.18632/aging.203584 (PMC8544304; doi:10.18632/aging.203584)
Supplement: Supplementary Table 2 [file aging-13-203584-s007.pdf]

SUPPLEMENTARY TABLE

Supplementary Table 2. The cutoff values of the 6 prognostic genes.

Gene	Cutoff
PDK4	13.37
MPP1	11.31
ASGR1	5.7
MS4A14	7.72
FCER1A	6.36
MX2	9.87

The first column was the name of the corresponding gene; the second column was the cutoff value of the corresponding gene.
